# Mouth-Breathing in Children, Particularly as a Result of Adenoids*Read at the Southern Section of the American Otological, Rhinological and Laryngological Association, Atlanta, March 28th.

**Published:** 1898-06

**Authors:** Arthur G. Hobbs

**Affiliations:** Atlanta, Ga.


ATLANTA
Medical and Surgical Journal.

VOL. XV. JUNE, 1898. NO. 4.

L. B. GRANDY, M.D., DUNBAR ROY, M.D., EDITORS.
M. B. HUTCHINS, M.D., BUSINESS MANAGER.

### ORIGINAL COMMUNICATIONS.

### MOUTH-BREATHING IN CHILDREN, PARTICULARLY AS A RESULT OF ADENOIDS.*

BY ARTHUR G. HOBBS, M.D.,
ATLANTA, GA.

That the nose was intended by nature only as an olfactory organ is the accepted belief of the laity. But, that so many of the profession should yet entertain the same idea is more to be wondered at. It would seem that many doctors have never taken the time to allow it to occur to them that olfaction is only a secondary function of the nose, and that the space allotted to this least important of the senses is confined to a very small part of this prominent feature.

The nose is intended for breathing through and because of this very important function and on account of its possible closure from accident, or otherwise, the mouth may be brought into service as an auxiliary in time of need, but only temporarily—indeed only as an auxiliary to respiration. As an automatic organ, there are but

* Read at the Southern Section of the American Otological, Rhinological and Laryngological Association, Atlanta, March 28th.

few in the body to compare with the work performed by the lining membrane of a healthy and natural nasal mucous membrane. It supplies the proper moisture to adapt the air to the lungs by secreting an immense amount of fluid for dry air to pass over, or a much less amount when the air is moist, thus automatically adapting itself to the humidity of the inspired air. As in the gills of a fish, nature has supplied a large superficial area of mucous membrane surface to this upper part of the respiratory tract—about thirty-six square inches as computed by Dr. Onodi. And Binz, Onodi and others have estimated that as much as two pints of fluid are secreted daily by this large surface when the air is devoid of humidity, in order to supply it with sufficient moisture to make it suitable to the parts below, where the mucous membrane is less active in its secretory function, and hence, less automatic in its response to the necessities of a uniform degree of humidity of the inspired air. The ciliated epithelium of the mucous surface of the lower air passages performs another function than that of active secretion, which is generally the office of a membrane with a squamous epithelium, such as we find the nasal and pharyngeal mucous membrane supplied with.

In mouth-breathing the inhaled air cannot pass over more than one-half of the moist surface that it does when inhaled through the nose, and this small surface is insufficient to impart the necessary moisture. Hence—and how natural—the parched mouth and dry throat of one who has breathed, during sleep, through nature's auxiliary, the mouth only. It would not be a stretch of the imagination should we reason by inference alone, without pausing to consider the many well-known and easily demonstrable results, how disastrous to the lower air passages a constant and continuous mouth-breathing would necessarily be to a growing child. But this is not all. The other one of the principal functions of the nasal membrane seems even more wonderful in its automatic action. It warms the inspired air when it is too cold for the lower air passages. The arterial circulation of the membrane becomes greatly accelerated by the inhalation of cold air, without reference to its secretory function, and automatically regulates its surface heat according to the temperature of the inspired air. When the air is dry and warm, its secretion alone is accelerated, and when it is cold and not devoid of humidity, the increased activity of the arterial circulation supplies more heat than fluid.

It would then be unnatural to expect an organ so delicately poised not to easily become unbalanced when it is so constantly and directly subjected to the many sudden changes of temperature and humidity. And how much more so when we remember that it has to bear the brunt, indirectly, of so many of nature's traversed laws through other, and even the most distant, parts of the body. Particularly should we be impressed with this fact when we remember that this organ is called upon to perform these automatic functions even in our climate with its almost daily changes of humidity and its great ranges of temperature.

It is not necessary to follow up the pathological results of this constant battle that never ceases, where in so many cases it finally fails, to preserve its physiological condition. Each recurrent attack adds to the effect produced before, ere nature has had the time or the opportunity to bring about the proper resolution. Hence the result is nasal stenosis from an hyperplasia or an hypertrophy of the turbinal membrane. Mouth-breathing naturally results, and it is fortunate that nature has provided this auxiliary in such cases of need. Yet this substitute may, on the other hand, be considered a misfortune to the growing child, when we think of the many results of a chronic stenosis. The circulation of the blood is interfered with, and hence the development of the child is perverted and retarded, the facial expression becomes unnatural, the palate becomes more arched, and the nose more flattened, together with many more results so well known to all who see these cases daily.

After childhood, and especially when adult life is reached, the space between the opposing mucous surfaces is so much greater that a stenosis from turbinal thickening alone rarely results in mouth-breathing during the waking hours, although the voice tone may be so much muffled as to suggest an adenoid or a polypus.

In children, the nasal stenosis which results from turbinal thickening has the additional factor of the undislodged secretions which can result only in mouth-breathing. But when due to this cause alone the history usually shows a short duration, since nature's efforts, assisted or not, are as a rule equal to the emergency of bringing about the proper restoration unless the recurrent attacks become too frequent.

But how different is the picture when adenoid growths fill this cavity and necessitate breathing through the mouth. The history of the case does not then tell of an acute, or a transitory condition. The child's pinched face, open mouth, voice tone, and general appearance of malnutrition would belie such a statement.

There are many cases that present themselves to us for relief, and too often with the vain hope of obtaining it. We occasionally have cases that we may selfishly wish had gone to some one else, knowing full well that neither could accomplish the end desired. A few of us have learned that we can assist nature to cure some of the cases sometimes, many of us think we can do so in some of the cases all of the time; but the majority of us have learned enough to know that we cannot cure all the cases all the time. But fortunately, when mouth-breathing in a child is caused by an adenoid, stenosis or a polypus, we know as confidently what the result of proper treatment will be in all such cases as we could know anything in surgery positively.

I will quote some lines from my paper describing a new instrument for the excision of adenoids: "A post-pharyngeal adenoid is as truly an overgrowth of Luscka's tonsil as is a wart an overgrowth of a papilla of the epidermis. The two differ in all respects only as the corium of the skin differs from the lymphoid cushion in the pharynx, whence each has its respective origin. The resemblance between them may be seen in the different degrees of consistency that each presents, due in both cases to the rapidity of growth, the age, and the peculiar condition of irritation to which either may have been subjected. While it is so-called hypertrophy, it is but little like the hypertrophies that we encounter elsewhere, particularly in children."

Nothing is definitely known as to the cause of adenoid growths, unless an hereditary tendency, as a predisposing cause, together with often-recurring colds as the excitant, can be regarded as a satisfactory etiology. They occur most frequently in children, and may disappear spontaneously when the subject has nearly or quite reached maturity, still it is not a rare occurrence to find them in young adults or even in those who have reached middle age.

The diagnosis is not difficult; indeed, by exclusion, the nature and character of the growth can be determined by digital examination as the surest means in children. The finger finds a mass in the superior pharynx that is yielding and gelatinous, with a furrowed surface that imparts a wormy feeling.

A glance at the child or even to hear his muffled tone is often all that is necessary to form a diagnosis. But the age should be considered and also the extent and character of the deafness if any; the open mouth with the vacant expression; the imperfect chest development; the ill-shaped nose with its small alæ; the inability to blow the nose, together with the history of snoring or difficult sleep-breathing. On the other hand it does not always follow that the child has adenoids because he snores and keeps his mouth open and is constantly enjoined by the mother and nurse to blow his nose, since a catarrhal tumefaction of the turbinals, enlarged tonsils or foreign bodies such as buttons, beans, etc., deliberately thrust into the nose by the child may have been the cause. In children it is not so necessary to exclude polypi or post-nasal abscesses, or bony growths, as these are rarely found at this time of life.

It would not be apropos in this paper to discuss the sequelæ of nasal stenosis in children farther than to mention the not unfrequent middle ear inflammation with its natural results of a rupture of the drum membrane, which in scorbutic children usually ends in chronic suppuration.

In any case the treatment that succeeds in restoring an opening to nature's normal breathing space which will enable the nose to perform its double function of supplying moisture and warmth to the inspired air accomplishes many good results that are oftentimes unexpected. The use of the finger-nail, or the gouge-shaped thimble is advocated by some, but should be resorted to only in urgent cases. Meyer, who has the honor of first having described adenoid vegetations, still resorts to the post-nasal curette in children. Voltolini seems to prefer the wire loop inserted beneath the palate, a very questionable procedure in children. The bullet forceps of Loenburg and Michael may be resorted to and safely used in many cases. Gleitzman and Elsburg's forceps are much more frequently used in England and on the continent than in this country. In most cases requiring an operation in little fellows who have to be held, as well as in many of those who are reasonable enough to accept the inevitable, I use my own forceps which have large fenestræ and small shanks. These large openings in the blades allow the soft mass to pass through which enables the operator to very rapidly make a number of grasps on the mass without having to withdraw the instrument. These forceps are equally as adaptable to operating when anesthesia is considered necessary.

I will not mention the many other instruments that have been and are now being used for the excision of adenoids. Notwithstanding their large number and variety no one instrument, or no one means of operating, has seemed to have gained any decided preponderance of favor. The very nature of these cases, as we find them in children, affording as they do so many ways of being reached, and capable of such brilliant results when successfully reached, is calculated to develop an individuality in the operator. I realize that I do not agree with most operators when I say that I have not found it necessary to extricate the whole mass, although I would consider it desirable. It has been my observation that a complete extirpation is rarely necessary since any remaining part of the growth atrophies when its greater portion has been removed and nasal-breathing has been reestablished.

A proper course of constitutional treatment is almost as essential as the surgical in a pale, anemic, mouth-breathing child with his ill-shaped nose and open mouth, pathetic expression of the eyes, muffled tone of the voice, etc. Such alteratives as the syrup of the iodide of iron, or iodine in some other acceptable form; the chlorides alone, or combined with cinchona in some form, or with the now generally known succus alterans, will produce as decided a tonic effect as the iodides and mercury do in syphilis. No class of patients is more satisfactory when we consider the almost certain good results that follow the proper surgical and constitutional treatment for mouth-breathing in children.

